# A multi-omics integrative analyses reveals *CYBB*-mediated apoptotic and immune dysregulation as a key target of berberine in diffuse large B-cell lymphoma

**DOI:** 10.3389/fphar.2026.1870435

**Published:** 2026-08-28

**Authors:** Jiewen Tan, Yunman Xu, Yueping He, Chang Chen, Jingwen Luo, Changxiu Zhang, Jinman Zhong, Dan Xiong

**Affiliations:** Department of Hematology, The Eighth Affiliated Hospital, Southern Medical University (The First People’s Hospital of Shunde, Foshan), Foshan, China

**Keywords:** apoptosis, berberine, CYBB, diffuse large B-cell lymphoma, immune microenvironment

## Abstract

**Background:**

*Berberine* has shown antitumor activity across multiple malignancies, yet its molecular targets and mechanisms of action in diffuse large B-cell lymphoma (DLBCL) remain incompletely defined. We integrated genetic causality, multi-omics data, and functional validation to identify berberine-related molecular pathways relevant to DLBCL.

**Methods:**

We identify bioinformatic screening and Mendelian randomization (MR) on 760 candidate berberine-related genes; 315 with adequate instruments underwent MR for causal links to DLBCL. Follow-up analyses included pathway enrichment, Protein-Protein Interaction (PPI) networks, machine-learning prognostic modeling, molecular docking and dynamics, single-cell RNA sequencing, cell–cell communication inference, bulk transcriptomic validation, and *in vitro* assays.

**Results:**

MR identified several berberine-related genes with putative causal associations with DLBCL. *ABCA1*, *ITK, MAPK3, TGM2, and DAPK1* were associated with decreased DLBCL risk, whereas *CYBB, RAC1, JUN, and CPT1A* were associated with increased risk. Enriched pathways encompassed B-cell receptor signaling, immune checkpoints, apoptosis, and MAPK signaling. Apoptosis pathway activity correlated with favorable prognosis across cohorts. Integrative analyses prioritized *CYBB* as a central hub. Molecular docking and dynamics indicated stable berberine–*CYBB* binding. Single-cell analyses showed *CYBB* + B cells had distinct apoptotic, immunoregulatory, and communication features, including BTLA–TNFRSF14 signaling. Bulk deconvolution linked high *CYBB* to worse prognosis, proliferation, and an immunosuppressive microenvironment. Functionally, *CYBB* knockdown reduced DLBCL cell viability and increased apoptosis; berberine inhibited growth dose-dependently, and cellular thermal shift assays supported berberine–*CYBB* target engagement in cells.

**Conclusion:**

This integrative study identifies berberine-related genes with potential causal relevance to DLBCL and prioritizes CYBB as a functionally relevant target linked to apoptosis, immune regulation, and prognosis, highlighting the potential involvement of berberine–*CYBB* axis underlying berberine activity in DLBCL, although rescue experiments are needed to confirm its specificity and relative contribution.

## 1 Introduction

Diffuse large B-cell lymphoma (DLBCL) is the most common subtype of non-Hodgkin lymphoma and exhibits pronounced molecular heterogeneity, diverse biological behavior, and variable clinical outcomes (Liu and Barta, 2019; Martelli et al., 2013). Transcriptomic and genomic profiling has established biologically distinct DLBCL subtypes driven by alterations in B-cell receptor signaling, immune regulation, and apoptosis-related pathways, which together shape disease progression and therapeutic response (Alizadeh et al., 2000; Chapuy et al., 2018; Schmitz et al., 2018). Despite the success of immunochemotherapy regimens such as R-CHOP, a substantial proportion of patients experience relapse or refractory disease, highlighting the need for additional molecularly informed therapeutic strategies.

Berberine, a naturally occurring isoquinoline alkaloid derived from medicinal plants such as *Coptis chinensis* Franch. and species of the genus *Berberis*, has demonstrated anticancer activity across multiple solid and hematological malignancies (Wang et al., 2020). Experimental studies suggest that berberine influences diverse biological processes, including cell proliferation, apoptosis, metabolic regulation, oxidative stress, and immune signaling (Liu et al., 2019; Tillhon et al., 2012; Xiong et al., 2022). However, most mechanistic insights are derived from *in vitro* or animal models, and how berberine-associated molecular programs relate to human cancer susceptibility and disease biology remains incompletely characterized. In particular, the extent to which genes linked to berberine-responsive pathways contribute to DLBCL risk or operate within specific cellular contexts of the tumor microenvironment has not been well defined.

A key challenge in translating such pharmacological observations into clinically meaningful insights lies in distinguishing causal disease drivers from secondary molecular changes. Mendelian randomization (MR) offers a genetic framework to address this issue by leveraging germline variants to infer causal relationships between gene expression and disease risk, thereby mitigating confounding and reverse causation (Davey Smith and Hemani, 2014). MR-based approaches have been increasingly applied to prioritize therapeutic targets in complex diseases (Schärfe et al., 2017; Zheng et al., 2020), including cancer, yet their application to natural compounds and DLBCL-relevant molecular pathways remains limited. Beyond tumor-intrinsic alterations, DLBCL progression is strongly influenced by the tumor microenvironment (TME). Single-cell transcriptomic studies have revealed marked heterogeneity among malignant B cells and infiltrating immune populations, uncovering cell-type-specific signaling programs and immune regulatory interactions that are not captured by bulk analyses (Roider et al., 2020; Steen et al., 2021). Immune regulatory pathways, including immune checkpoint signaling such as the BTLA–TNFRSF14 axis, have been implicated in apoptosis regulation, immune modulation, and lymphoma progression (Šedý et al., 2020; Watanabe et al., 2003). Integrating genetic causal inference with single-cell–resolved analyses therefore provides an opportunity to clarify how candidate genes linked to berberine-associated pathways may operate within specific cellular contexts in DLBCL.

In this study, we applied an integrative framework combining large-scale bioinformatic screening, Mendelian randomization, pathway and network analyses, single-cell transcriptomics, and experimental validation to prioritize berberine-associated genes with potential causal relevance to DLBCL. This strategy aims to elucidate the molecular and cellular mechanisms through which berberine-related pathways may influence lymphoma biology, with particular emphasis on apoptosis, immune regulation, and tumor–microenvironment interactions.

## 2 Methods

### 2.1 Data sources and preprocessing

We retrieved publicly deposited bulk transcriptomic data to investigate gene expression patterns and clinical relevance in DLBCL. Tumor sample sets from the GEO database, including GSE10846 (Lenz, Wright, Dave, et al., 2008) (n = 422), GSE11318 (Lenz, Wright, Emre, et al., 2008) (n = 203), GSE181063 (Lacy et al., 2020) (n = 1,310), GSE53786 (Scott et al., 2014) (n = 119), and GSE23501 (Shaknovich et al., 2010) (n = 69), were used for differential expression and survival analyses. Furthermore, a machine learning model (see Section 2.5) was constructed using combined datasets of GSE10846 (Lenz, Wright, Dave, et al., 2008), GSE11318 (Lenz, Wright, Emre, et al., 2008), GSE181063 (Lacy et al., 2020), GSE53786 (Scott et al., 2014), GSE23501 (Shaknovich et al., 2010), GSE57611 (Scholtysik et al., 2015), GSE69051 (Sha et al., 2015), and GSE87371 (Dubois et al., 2017) to further identify molecular features and prognostic patterns associated with *CYBB*. In addition, we also retrieved Single-cell data from four DLBCL samples in the GSE182434 (Steen et al., 2021) dataset were selected for analysis.

Berberine-related candidate target genes were collected from five publicly available target-gene resources, including BATMAN-TCM, GeneCards, PharmMapper, SwissTargetPrediction, and the Comparative Toxicogenomics Database (CTD). To maximize coverage, all publicly available berberine-related target genes from these databases were retrieved regardless of disease type, tissue, or cell line. No DLBCL-specific or cell line-specific filtering was applied at this stage. Candidate genes obtained from each database were standardized to official gene symbols, and duplicate genes across databases were removed. After integration and deduplication, a total of 760 unique berberine-related candidate genes were identified and used as the initial target-gene set (Supplementary Table S1).

### 2.2 Mendelian randomization

A two sample MR was used to examine the potential causal relationship between target genes’ expressions and DLBCL. These MR analyses were conducted using the TwoSampleMR R package (v0.5.7) (Hemani et al., 2017). Instrumental variables (IVs) were selected from eQTL data in the eQTLGen study (n = 31,684) (Võsa et al., 2021), with significance threshold at p < 5 × 10^−8^. Confounding variants were excluded via PhenoScanner, and linkage disequilibrium parameters were set to *r* ^2^ < 0.01 (within a 500 kb range). We obtained the GWAS summary statistics of DLBCL (outcome) from FinnGen study (Kurki et al., 2023), in which 1,010 DLBCL cases and 287,137 controls were included. To assess the robustness of our results, we applied five commonly used MR methods in this study, including Inverse Variance Weighted (IVW), MR-Egger, Weighted Median, Simple Mode, and Weighted Mode (Hemani et al., 2017): Positive results were defined based on the following criteria: (1) IVW with a p-value <0.05; (2) consistent effect direction across all five methods; (3) no significant heterogeneity (IVW Cochran’s Q test p > 0.05); (4) absence of horizontal pleiotropy (MR-PRESSO and Egger intercept tests with p > 0.05); (5) robustness in leave-one-out sensitivity analysis; (6) F-statistic >10 to exclude weak instrument bias; and (7) Steiger test p < 0.05 to confirm no reverse causality.

### 2.3 Functional enrichment

The R package “clusterProfiler” was utilized to conduct GO, KEGG, and Hallmark pathway enrichment analyses. These enrichment analyses were conducted separately by upregulated, downregulated and all differential genes. Genes with a p-value < 0.05 was considered significantly enriched, and the top 15 pathways were selected for visualization (or all pathways if fewer than 15).

### 2.4 Protein-Protein Interaction (PPI), molecular docking, and molecular dynamics simulation

PPI network analysis was performed for berberine-associated target genes prioritized as candidate genes potentially related to DLBCL. The interaction network was constructed using the STRING database v12.0 with *Homo sapiens* selected as the reference organism. To retain functionally associated interactions, edges with a combined score ≥0.15 were included. To reduce the influence of weak interactions driven only by text mining, each retained edge was further required to be supported by at least two independent STRING evidence types.

To evaluate potential binding between berberine and proteins encoded by prioritized genes, molecular docking analyses were conducted. The chemical structure of berberine was obtained from PubChem, and structures of targeted proteins were retrieved from the RCSB Protein Data Bank. Specifically, Protein structures were extracted using Biopython (v1.8.3), with file formats being converted using OpenBabel (v3.1.1).

Molecular docking was performed using the CB-Dock2 (Liu Y et al., 2022) web server. Both pocket-based docking and blind docking were considered. For pocket-based docking validation, the co-crystallized ligand was extracted from the protein structure and redocked into its native binding pocket using the same docking workflow. The redocked ligand pose was aligned with the crystallographic ligand conformation, and the root-mean-square deviation (RMSD) between the two conformations was calculated. An RMSD value <2.0 Å was considered indicative of acceptable reproduction of the experimentally observed binding pose. The calculated RMSD was 1.33 Å, demonstrating that the docking protocol reliably reproduced the native ligand-binding conformation. Berberine was then docked into the validated pocket. Blind docking was additionally performed without restricting the ligand to the native ligand pocket to explore other potential binding regions. Docking scores were interpreted using ≤−7.0 kcal/mol as the criterion for strong predicted binding affinity.

Molecular dynamics simulation was further performed to evaluate the stability of the predicted berberine-protein complex. Simulations were conducted using GROMACS 2022 (Abraham et al., 2015). The protein was modeled using the AMBER14SB force field, and berberine was parameterized using the GAFF2 force field. The complex was solvated using the TIP3P water model, with the simulation box extending 1 nm from the protein surface, and the system was neutralized with ions. Long-range electrostatic interactions were treated using the particle mesh Ewald method with a 1-nm cutoff. Bond constraints were applied using the SHAKE algorithm (Ryckaert et al., 1977), and the integration time step was set to 1 fs. Simulations were performed under the NPT ensemble at 310 K for a total duration of 100 ns. Energy minimization included 3,000 steps of steepest descent followed by 2,000 steps of conjugate-gradient minimization, followed by staged restrained equilibration before the production simulation.

Trajectory analyses were conducted to assess the stability and conformational dynamics of the complex, including root-mean-square deviation, radius of gyration, solvent-accessible surface area, hydrogen-bond number, root-mean-square fluctuation, and free-energy landscape analysis. The time required for RMSD stabilization was used to define the equilibration period, and the remaining trajectory was used as the stable sampling phase for downstream interpretation.

### 2.5 Machine learning–based prognostic model construction

We applied 10 machine learning algorithms to construct models for the prognosis of DLBCL. A total of 101 combinations were considered during the parameter tuning process. Specifically, we used packages “glmnet” (v4.1–8) for Lasso, Ridge, and Elastic Net regression; “survival” (v3.5–3) for stepwise Cox regression; “survivalSVM” (v0.05) for survival Support Vector Machine; “CoxBoost” (v1.5) for Cox boosting; “superpc” (v1.12) for supervised principal component analysis; “plsRcox” (v1.77) for Cox partial least squares regression; “randomForestSRC” (v3.23) for random survival forests; and “gbm” (v2.19) for gradient boosting machines. We built the prognostic model following four steps: (1) In the GSE10846 cohort, using berberine-targeted DLBCL causal gene expression as input variables and survival data as outcome variables, 101 algorithm combination prediction models were built based on a leave-one-out cross-validation (LOOCV) framework to minimize overfitting risks; (2) Models were validated across seven independent datasets (GSE181063 [Lacy et al., 2020], GSE53786 [Scott et al., 2014], GSE57611 [Scholtysik et al., 2015], GSE69051 [Sha et al., 2015], and GSE87371 [Dubois et al., 2017]. GSE23501 [Shaknovich et al., 2010], GSE11318 [Lenz, Wright, Emre, et al., 2008]); (3) Harrell’s C-index was calculated for each model to evaluate the performance of prediction; (4) the model with the highest and most stable average C-index across training and validation sets was selected. We further grouped included patients into high- and low-risk groups based on the estimated median risk score. Prognostic performance was further evaluated across clinical subgroups. A web-based prediction platform was implemented using the Shiny package.

### 2.6 Single-cell RNA sequencing analysis

R package Seurat (v4.1.1) (Hao et al., 2024) was used to process obtained single-cell sequencing data in line with standard protocol. Cells with mitochondrial content >10%, hemoglobin content >5%, or expressed gene counts <200 or >30,000 were filtered out. To address technical artifacts, DoubletFinder R package (McGinnis et al., 2019) was applied for doublet exclusion. The expected doublet rate was set to 7.5%, estimated according to the 10x Genomics cell-recovery guideline for approximately 12,000 captured cells. The pK parameter was optimized using the paramSweep function, and pK = 0.09 was selected based on the maximum doublet-classification performance.

“RunHarmony” function in the harmony R package (Korsunsky et al., 2019) was used to mitigate batch effects. Specifically, harmony integration was performed using “dataset” as the batch variable, and downstream clustering was conducted based on the Harmony-reduced dimensions. The analysis focused on 2,000 highly variable genes. Linear dimensionality reduction was performed using principal component analysis (PCA). The first 20 PCs were retained because they provided stable clustering and clear separation of major immune and B-cell populations according to canonical marker expression. Clustering was performed using the FindClusters algorithm with resolution = 0.6, which similarly provided interpretable cluster separation consistent with known cell-type markers. Nonlinear embedding was performed using UMAP with the RunUMAP function.

The identified cell clusters were then annotated according to the expressions of marker genes via accessing the CellMarker 2.0 database. B cells were identified using canonical B-cell markers, and DLBCL-associated B-cell populations were analyzed after excluding non-B immune cells. Because clonal BCR sequencing or single-cell CNV confirmation was not available, these cells were described as DLBCL-associated B cells rather than definitively malignant B cells where appropriate. For *CYBB*-based stratification, CYBB-detected B cells were defined as B cells with detectable CYBB expression (>0), whereas CYBB-undetected B cells were defined as B cells in which no CYBB transcript was detected (=0) in the analyzed scRNA-seq data. Because zero measurements in scRNA-seq may arise from limited transcript capture, stochastic expression, technical dropout, or biological non-expression, the term “CYBB-undetected” does not imply definitive biological absence of CYBB expression. A sensitivity analysis was also performed using the median *CYBB* expression level to classify B cells into *CYBB*-high and *CYBB*-low groups.

We further constructed pseudotime trajectories based on cellular gene expression profiles using Monocle (v2.28.0) (Qiu et al., 2017). Before pseudotime analysis, B-cell subsets were further filtered to reduce potential T/NK-cell contamination or doublets. Cells expressing canonical T-cell markers CD3D or CD3E were removed, and the retained cells were verified based on canonical B-cell marker expression and absence of T-cell marker expression at the single-cell level. Apoptosis-related genes and BCL2 expression were further examined along the pseudotime trajectory to characterize apoptosis-associated transcriptional changes. Functional enrichment analyses, including GO, KEGG, and HALLMARK gene set analysis (Yu et al., 2012), were performed on these upregulated genes. As stated above, these enrichment analyses were conducted with the R package “clusterProfiler” (v4.8.2). The downstream potential interactions between different cell types were further explored using the CellChat (v2.1.2) (Jin et al., 2025) algorithm. Differential enrichment, cell-cell communication, and pseudotime analyses were repeated in the *CYBB*-high and *CYBB*-low sensitivity analysis to evaluate robustness to the *CYBB* stratification threshold.

### 2.7 Immune infiltration and Bayesian deconvolution analysis

Immune infiltration analysis was performed to assess the association between CYBB expression and the DLBCL tumor immune microenvironment. Bulk immune-deconvolution analyses were conducted mainly in the GSE10846 cohort. Cellular infiltration levels were estimated using CIBERSORT v4.2.3, xCell v1.1.0, and MCPcounter. These algorithms were applied in parallel to evaluate immune-cell infiltration patterns across different deconvolution frameworks. The ESTIMATE R package v1.0.13 was used to calculate Stromal Score, Immune Score, ESTIMATE Score, and tumor purity. In addition, ssGSEA was performed using the GSVA R package v1.48.3 to evaluate immune-related functional signatures and pathway-level activity. For immune-infiltration and pathway-score analyses, samples were stratified into *CYBB*-high and *CYBB*-low groups using median *CYBB* expression. These analyses were performed to assess whether *CYBB* expression was associated with immune-cell infiltration, immune functional states, and tumor purity, and were interpreted together with single-cell cell–cell communication results to evaluate *CYBB*-associated immune patterns across transcriptomic scales.

BayesPrism (Chu et al., 2022) was further used to infer cell-state proportions and cell-type-specific gene-expression profiles from bulk transcriptomic data, using patient-derived single-cell RNA-seq profiles as the reference. Single-cell-derived expression profiles were used as priors, and Bayesian inference with Gibbs sampling was applied to estimate posterior cell-state proportions for each bulk sample. Deconvolution was performed independently for each sample and was not affected by downstream group balance. For analyses based on inferred CYBB-positive B-cell proportions, samples were stratified using the cutoff strategy described in Section 2.8. Sensitivity analyses using alternative cutoff strategies were performed to evaluate robustness.

### 2.8 Survival and cutoff determination

Survival analyses were performed to evaluate the prognostic relevance of gene expression levels or calculated risk scores. For Kaplan-Meier survival visualization, optimal cutoff values were determined using the surv_cutpoint function in the training cohort. The resulting cutoff was fixed and applied to validation cohorts without cohort-specific re-optimization. Because surv_cutpoint identifies the threshold that maximizes the log-rank statistic, cutoff-based analyses were considered exploratory; no Lausen-Schumacher correction was applied.

Patients were categorized into high- and low-expression or high- and low-risk groups according to the predefined optimized cutoff, and survival differences were assessed using Kaplan-Meier curves and log-rank tests. For downstream comparative analyses, including immune infiltration and pathway-score analyses, median dichotomization was used to define high- and low-expression groups. Continuous gene expression levels or risk scores were used for formal prognostic inference where applicable.

### 2.9 *In vitro* experimental validation

All *in vitro* experiments were performed using at least three independent biological replicates. The exact number of replicates for each experiment is reported in the corresponding figure legends.

#### 2.9.1 Cell lines and culture

The human DLBCL cell lines Ly8 and U2932 were acquired from the Cell Resource Center of the Shanghai Institutes for Biological Sciences (Chinese Academy of Sciences, Shanghai, China). These cells were maintained in RPMI 1640 medium supplemented with 10% fetal bovine serum (FBS) and 1% penicillin/streptomycin (100 U/mL), all sourced from Life Technologies (Grand Island, NY, United States, and Monza, Italy). Incubation was performed at 37 °C in a humidified environment containing 5% CO_2_.

#### 2.9.2 Cell transfection

Ly8 and U2932 cells were seeded at a density of 2 × 10^5^ cells/mL and incubated overnight. Cells were then transfected with human CYBB-targeting siRNA (si-CYBB) or a non-targeting negative control (si-NC). These siRNAs were obtained from Sangon Biotech (Shanghai, China), and their specific sequences are detailed in Supplementary Table S2. We utilized Lipofectamine 6,000 (Beyotime Biotechnology, Shanghai, China) for the lipid-mediated delivery, adhering to the manufacturer’s protocol. The growth medium was refreshed 6 h post-transfection. To determine the efficiency of the knockdown, qRT-PCR was conducted at 48–72 h following treatment.

#### 2.9.3 qPCR analysis of gene expression

Total RNA was extracted using the TIANGEN DP405-02 RNA Extraction Kit. mRNA levels were detected using the SYBR Green qPCR method (TaKaRa RR82LR). Primer sequences are listed in Supplementary Table S3.

#### 2.9.4 Western blot analysis

Total protein was extracted from cell lysates and measured via the BCA method (Biosharp, BL521A). We separated the protein samples on 10% SDS-PAGE gels and transferred them onto PVDF membranes. After blocking with 5% skim milk for 2 h at room temperature, the blots were incubated overnight at 4 °C with anti-CYBB (Proteintech, 19013-1-AP, 1:1,000) and anti-β-Actin (Proteintech, 66009-1-Ig, 1:5,000), followed by HRP-conjugated secondary antibodies for 1 h. Protein expression levels were analyzed using a Tanon detection system (Shanghai, China).Western blot experiments were independently repeated using separate biological samples, with the number of replicates specified in the corresponding figure legends. Complete, uncropped immunoblot images are provided in the Supplementary Material.

#### 2.9.5 CCK-8 cell viability assay

Ly8 and U2932 cells were seeded into 96-well plates (10,000 cells/well) and treated with si-NC/si-*CYBB* or varying drug concentrations. Cell viability was assessed using the CCK-8 kit (Beyotime, C0037).

#### 2.9.6 TUNEL assay

Apoptosis levels in Ly8 and U2932 cells were detected using a TUNEL assay kit, following the manufacturer’s instructions. Green TUNEL-positive nuclei were observed and captured using a fluorescence microscope. Image J software was applied to analyze each image and calculate the proportion of apoptotic cells. To minimize the influence of background fluorescence and low-intensity nonspecific signals, only cells with TUNEL fluorescence intensity above the predefined threshold of >30 grayscale units were counted as TUNEL-positive cells. Three randomly selected independent fields were analyzed for each group. The ratio of TUNEL-positive cells was calculated as: (Number of TUNEL-positive cells/Total number of cells) × 100%.

#### 2.9.7 Cellular thermal shift assay (CETSA)

To assess protein stability, logarithmic-phase DLBCL cells were seeded in 15 cm plates and incubated for 12 h with 55 μM berberine. Following incubation, the cells were collected and suspended in PBS supplemented with protease inhibitors. Aliquots of the cell suspension were transferred to PCR tubes and heated at designated temperatures for 3 min using a Veriti thermal cycler, then cooled at room temperature for 3 min. After three liquid nitrogen freeze–thaw cycles to ensure lysis, the samples were centrifuged (13,000 rpm at 4 °C for 10 min). The soluble protein fractions were then harvested for downstream immunoblotting. Similarly, the CETSA experiment was independently performed three times using separate biological replicates (n = 3). Complete, uncropped CETSA immunoblot images from the independent experiments are provided in the Supplementary Material.

### 2.10 Statistical analysis

All statistical evaluations were performed in the R environment (v4.0.5). To assess differences in continuous variables across two groups, we utilized Mann–Whitney U tests, while Kruskal–Wallis tests were applied for comparisons involving three groups. Categorical variable distributions were evaluated via Chi-square analysis. In all instances, a two-tailed p < 0.05 was the threshold for statistical significance.

## 3 Results

### 3.1 Identification of berberine target genes via Mendelian randomization

**FIGURE 1:**
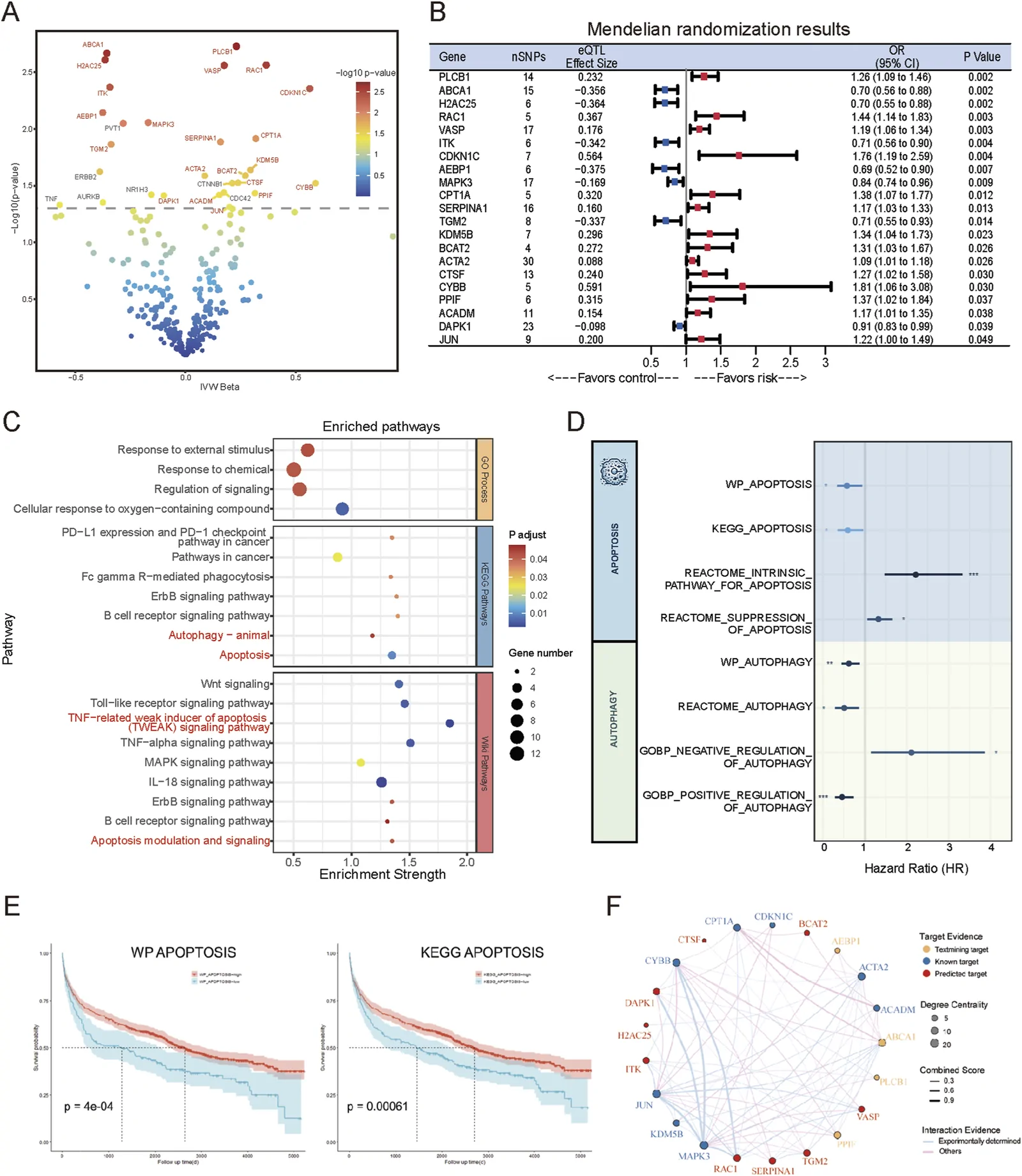
Screening and functional analysis of berberine target genes. **(A)** Volcano plot displaying the relationship between IVW Beta values and -Log10 (p-value); each dot represents a gene and its effect size and statistical significance related to DLBCL. **(B)** Forest plot presenting the Odds Ratios (OR) of individual genes affecting DLBCL risk. **(C)** Pathway enrichment analysis showing pathways significantly affected by Berberine target genes. **(D)** Forest plot displaying the association between autophagy and apoptosis pathway activity scores and DLBCL patient prognosis. **(E)** Kaplan-Meier survival curves stratified by high and low WP (Left) and KEGG (Right) APOPTOSIS signaling pathway activity, respectively. **(F)** Protein-Protein Interaction (PPI) network highlighting core nodes with the highest degree centrality (*MAPK3, JUN*, and *CYBB*).

After filtering for instrument availability (≥3 SNPs), 315 genes were taken forward for Mendelian randomization (MR). MR identified 21 genes with evidence of association between genetically predicted expression and DLBCL risk (Figure 1A), and Steiger testing did not support reverse directionality for these genes (Supplementary Figure S1A–D). Across the 21 genes, higher predicted expression of *ABCA1, H2AC25, ITK, AEBP1, MAPK3, TGM2,* and *DAPK1* was associated with lower DLBCL risk, whereas higher predicted expression of *PLCB1, RAC1, VASP, CDKN1C, CPT1A, SERPINA1, KDM5B, BCAT2, ACTA2, CTSF, CYBB, PPIF, ACADM*, and *JUN* was associated with higher risk (Figure 1B). Pathway enrichment of candidate genes highlighted immune and cancer-relevant pathways, including PD-1/PD-L1 signaling, B-cell receptor signaling, apoptosis, and MAPK signaling (Figures 1C,D). In GSE181063, pathway-level scoring suggested that apoptosis-related activity was associated with clinical outcomes, with higher activity of apoptosis gene sets corresponding to more favorable survival (Figure 1E). Several MR-prioritized genes also showed associations with survival in bulk cohorts (Supplementary Figure S2) PPI analysis identified *MAPK3*, *JUN*, and *CYBB* as highly connected nodes (Figure 1F), with detailed interaction scores and evidence types provided in Supplementary Table S4. To construct a robust prognostic model, we employed a permutation combination of 10 feature selection strategies and 11 machine learning algorithms, generating 101 candidate models evaluated via LOOCV. The results showed that the forward stepwise Cox model achieved the best discriminatory performance (mean C-index = 0.612). This model demonstrated stable prognostic discrimination across subgroups stratified by age (<60 vs. ≥60 years), Ann Arbor stage (I-II vs. III-IV), and IPI score (low vs. high risk), indicating strong clinical generalizability (Supplementary Figure S3).

### 3.2 Molecular simulation of berberine and CYBB targeted binding

**FIGURE 2:**
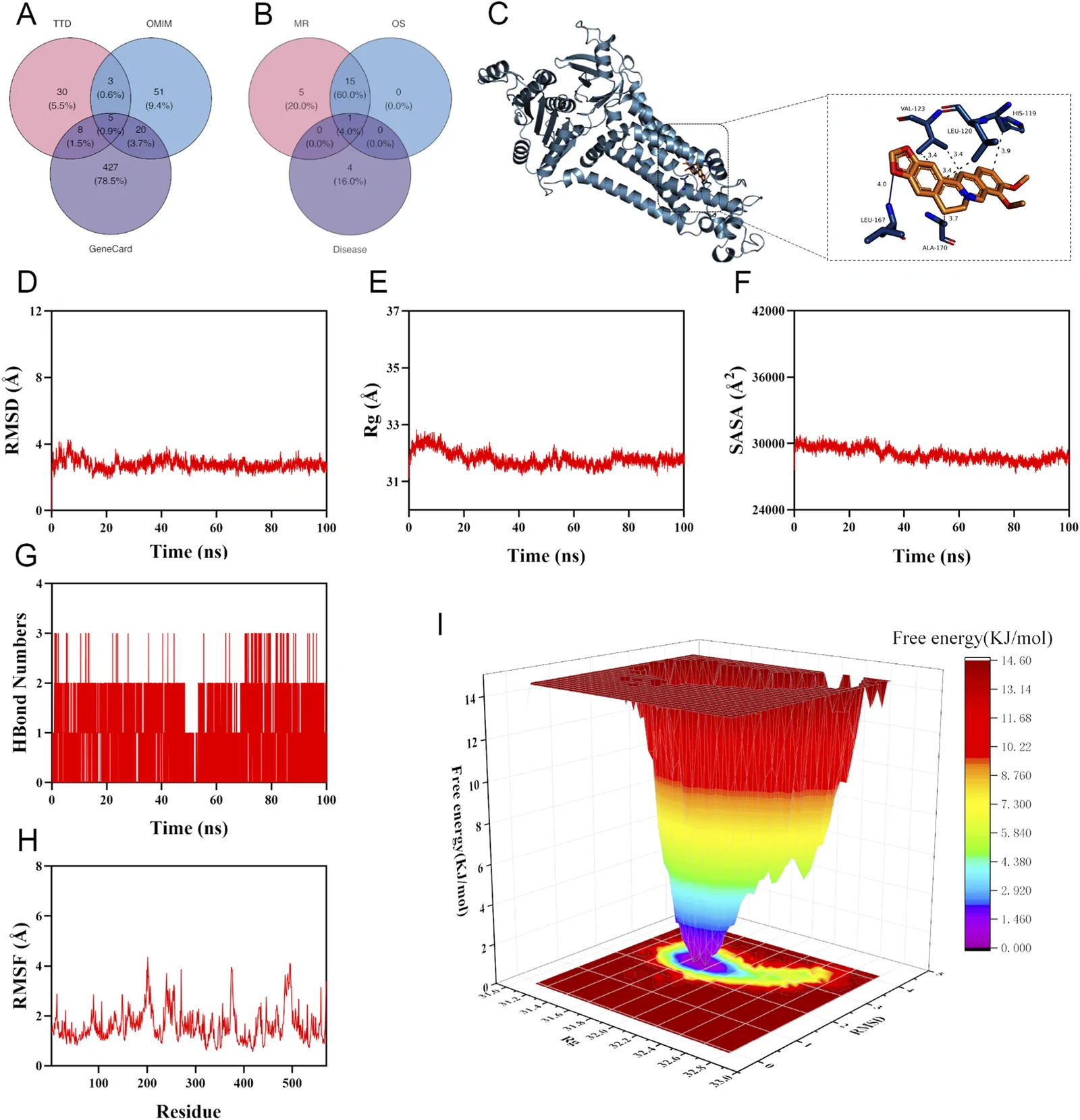
Computational simulation of berberine targeting *CYBB* for DLBCL treatment. **(A)** Screening of DLBCL-related genes from TTD, OMIM, and GeneCards. **(B)** Intersection of prognosis, causal association, and DLBCL disease database genes to identify *CYBB* as the core Berberine target gene. **(C)** Molecular interaction of Berberine within the *CYBB* binding pocket. In the two-dimensional interaction diagram, the blue solid line denotes a putative hydrogen bond, whereas the dotted lines denote predicted hydrophobic interactions. The numbers indicate the corresponding interaction distances in angstroms. **(D)** RMSD values of the protein-ligand complex over time. **(E)** Rg values of the protein-ligand complex over time. **(F)** SASA values of the protein-ligand complex over time. **(G)** Hydrogen bond (HBonds) values of the protein-ligand complex over time. **(H)** RMSF values of the protein-ligand complex. **(I)** Free Energy Landscape (FEL) plot.

*CYBB* was prioritized as a core target gene for berberine regulation of DLBCL (Figure 2A). To prioritize a candidate gene for downstream analyses, we intersected multiple evidence layers, including prognostic associations, MR identified genes, and disease-gene resources (Figure 2B). Molecular docking studies demonstrated a strong binding affinity (−9.1 kcal/mol) between *berberine* and *CYBB*. In the predicted binding pose, berberine occupied a putative CYBB binding pocket and interacted with several surrounding residues (Figure 2C). Specifically, LEU167 formed a putative hydrogen bond with berberine at a distance of approximately 4.0 Å, whereas HIS119, LEU120, VAL123, and ALA170 formed predicted hydrophobic contacts at distances ranging from 3.4 to 3.9 Å.

Molecular dynamics simulations indicated that the complex reached a relatively stable regime over the simulation window, with RMSD stabilizing after approximately 25 ns and modest variation in Rg and SASA (Figures 2D–F). Hydrogen-bond analysis showed intermittent bonding throughout the trajectory (Figure 2G), and RMSF suggested limited fluctuation in residues within the predicted binding region (Figure 2H). The FEL, based on a 2D projection of RMSD and Rg, revealed that the system primarily occupied low-energy conformational regions, with energy decreasing along a red-to-blue gradient, further corroborating the thermodynamic advantage of this binding (Figure 2I). These computational biology evidence elucidate the molecular mechanism by which berberine inhibits *CYBB* function by “locking” its conformation at the atomic scale, providing a structural basis for understanding its mechanism of action.

### 3.3 Single-cell analysis of berberine targeting CYBB in B cells

**FIGURE 3:**
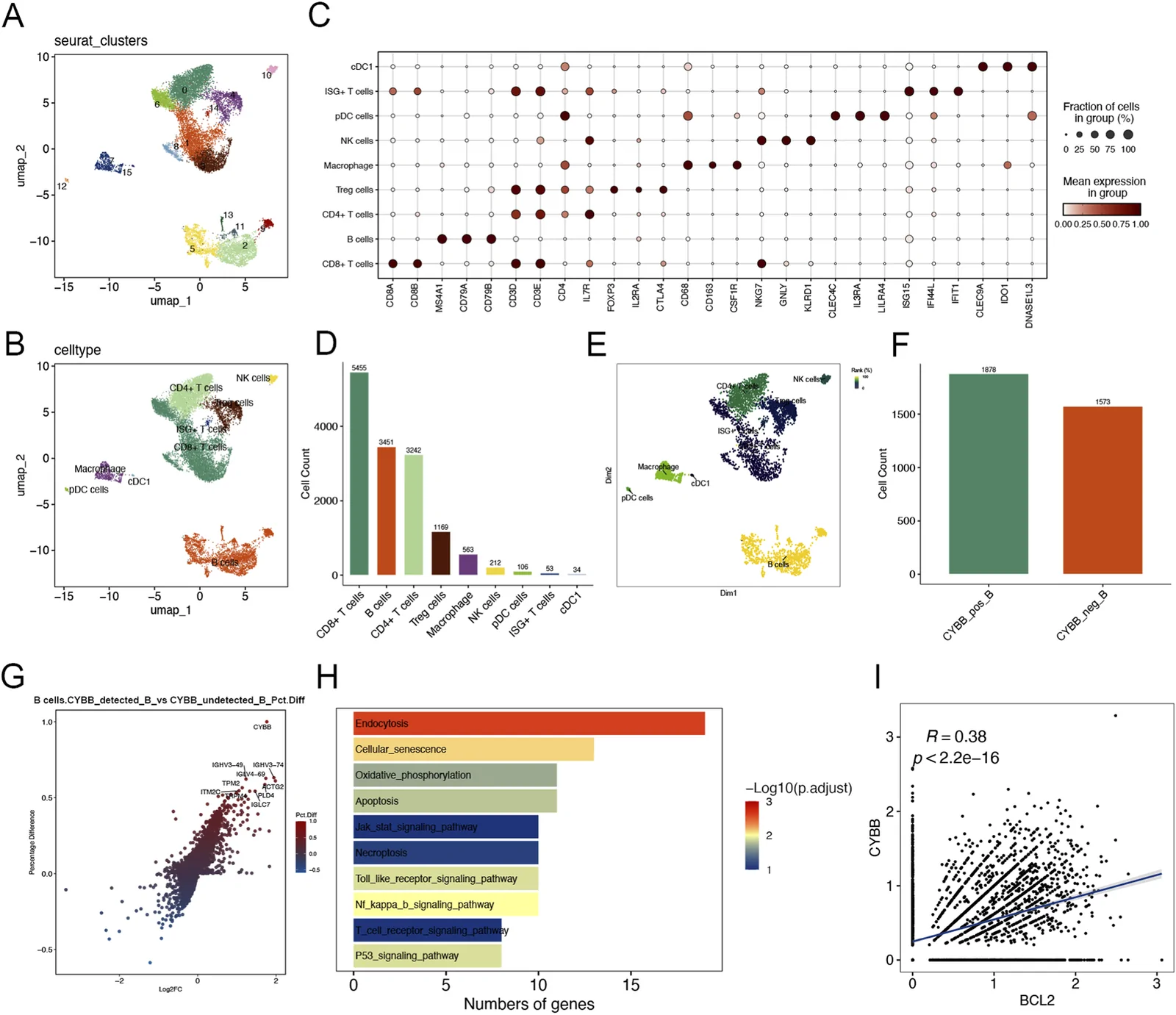
DLBCL Single-cell Atlas and functional characterization of *CYBB*-Positive/Negative B Cells. UMAP visualization for unsupervised clustering analysis; each dot represents an individual cell. **(A)** Colored by cell cluster. **(B)** Colored by cell type. **(C)** Display of characteristic marker genes for each cell type. **(D)** Cell counts for each cell type. **(E)** ScRank analysis identifying the action of Berberine across different cell types via the *CYBB* target. **(F)** Cell counts of *CYBB*_pos_B and *CYBB*_neg_B cells. **(G)** Volcano plot of differential genes between *CYBB*_pos_B and *CYBB*_neg_B cells. **(H)** KEGG enrichment analysis of upregulated genes in *CYBB*_pos_B vs. *CYBB*_neg_B cells. **(I)** Correlation analysis between *CYBB* and *Bcl2* expression levels.

Using single-cell data from GSE182434, we conducted dimensionality reduction and clustering analyses. Overall, we identified 16 distinct clusters of cells (Figure 3A), which were further reclassified into nine cell types (Figures 3B,C): NK cells, CD4^+^ T cells, Treg cells, ISG + T cells, CD8^+^ T cells, Macrophages, cDC1 (conventional type 1 dendritic cells), pDC cells (plasmacytoid dendritic cells), and B cells, based on expression of marker genes. Among them, CD8^+^ T cells were the most abundant (n = 5,455), while cDC1 cells were the least abundant (n = 34) (Figure 3D). To evaluate potential cellular targets of Berberine, we performed ScRank analysis and found that its action was primarily concentrated in B cells (Figure 3E). We further subdivided B cells into *CYBB*_pos_B (n = 1,878) and *CYBB*_neg_B (n = 1,573) subsets (Figure 3F). To elucidate the functional differences between these subpopulations in DLBCL, differential expression analysis was conducted and identified 1,087 DEGs, including 522 upregulated and 565 downregulated genes (Figure 3G). Functional enrichment analysis of the upregulated genes showed enrichment primarily in Apoptosis, JAK-STAT signaling pathway, Necroptosis, Toll-like receptor signaling pathway, NF-κB signaling pathway, T cell receptor signaling pathway, and P53 signaling pathway (Figure 3H). Additionally, *CYBB* expression was significantly positively correlated with the key apoptotic gene *BCL2* (Figure 3I), suggesting that *CYBB* may participate in DLBCL development by regulating apoptotic pathways.

### 3.4 Pseudotime analysis of B cells

**FIGURE 4:**
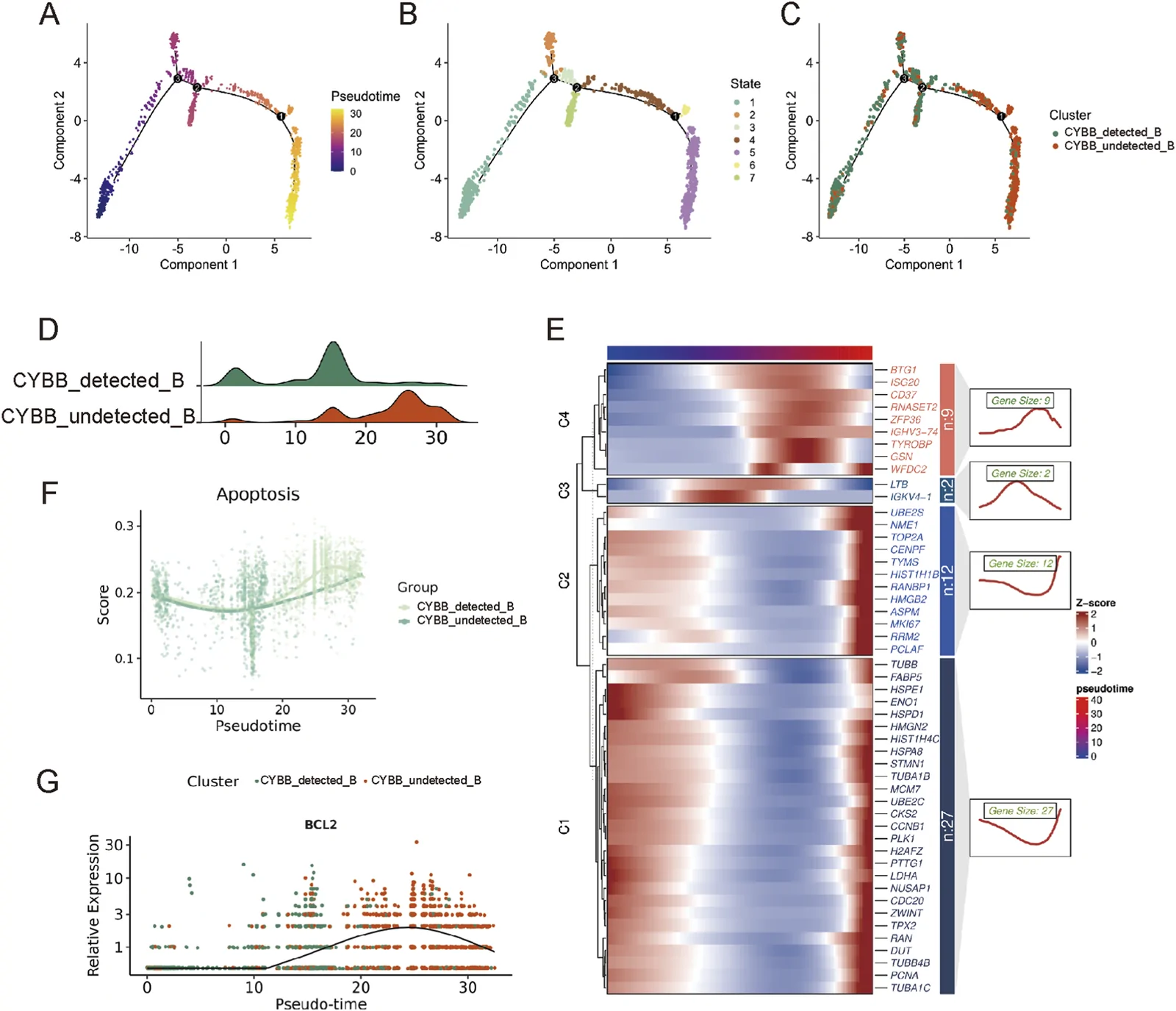
B cell pseudotime analysis. **(A)** Cell trajectory (colored by pseudotime order). **(B)** Cell differentiation process colored by state. **(C)** Cell subpopulation grouping colored by type. **(D)** Ridge plot of differentiation trajectories for different B cell subpopulations. **(E)** Heatmap showing the dynamic changes of branch-dependent gene expression along pseudotime. **(F)** KEGG enrichment results for Cluster 3 genes. **(G)** BCL2 expression dynamics along the B-cell pseudotime trajectory, colored by CYBB status. The black curve denotes the fitted expression trend.

To investigate the dynamic evolution mechanism of B cells, pseudotime trajectory analysis was performed (Figure 4A). The inferred B-cell trajectory was divided into nine distinct states (Figure 4B). Both *CYBB*-negative B cells (*CYBB*_neg_B) and *CYBB*-positive B cells (*CYBB*_pos_B) were distributed across all time points of the pseudotime differentiation trajectory (Figure 4C). The ridge plot further showed the distribution of B-cell subsets along pseudotime (Figure 4D). CYBB_neg_B cells were distributed across the entire inferred transcriptional trajectory, whereas CYBB_pos_B cells were primarily concentrated at later pseudotime states. These results suggest that CYBB_neg_B and CYBB_pos_B cells occupy distinct transcriptional states along the pseudotime axis, rather than demonstrating a definitive lineage differentiation process.

BEAM analyses further identified 50 branch-dependent genes that may play critical roles in regulating cell differentiation (Figure 4E). These genes were further categorized into four modules (clusters) that with distinct trends along the pseudotime trajectory. Herein, Cluster 1 expression decreased then increased; Cluster 2 increased then decreased; Cluster 3 showed an increasing trend; and Cluster 4 increased then decreased. Notably, Cluster 3 was primarily enriched in processes such as Apoptosis, Natural killer cell-mediated cytotoxicity, T cell receptor signaling pathway, and NF-kappa B signaling pathway (Figure 4F). Because pseudotime analysis reflects transcriptomic ordering rather than confirmed lineage commitment, these results indicate that apoptosis- and immune-related programs are associated with later CYBB-enriched transcriptional states, rather than proving that CYBB_neg_B cells differentiate into CYBB_pos_B cells.

To further clarify the apparent coexistence of apoptosis pathway enrichment and adverse CYBB-related features, we examined apoptosis-related genes contributing to the enrichment signal. The apoptosis pathway enrichment in CYBB_pos_B cells involved not only apoptosis-related signaling components but also anti-apoptotic or survival-associated genes, including *BCL2, BCL2A1, and BIRC3*. These genes were positively associated with CYBB expression. In addition, *BCL2* expression increased along pseudotime in a pattern broadly consistent with CYBB expression (Figure 4G). These findings suggest that CYBB_pos_B cells may represent a transcriptional state characterized by apoptosis-related pathway activity together with anti-apoptotic gene expression, consistent with an apoptosis-resistant or stress-adaptive phenotype rather than effective apoptotic execution.

### 3.5 Cell-cell communication

**FIGURE 5:**
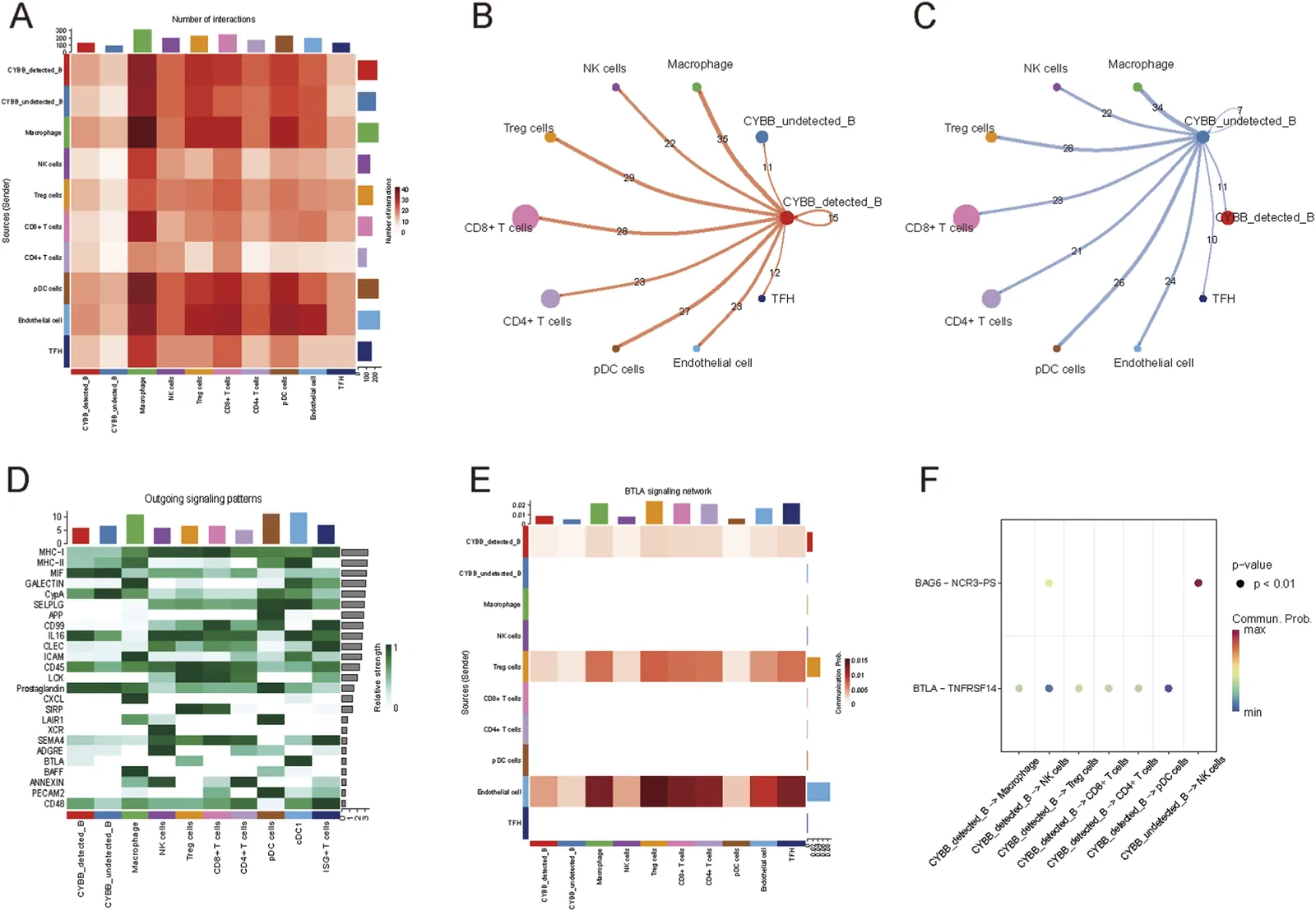
Cell-cell communication. **(A)** Heatmap displaying the total number of interactions between cell types. **(B)** Communication network diagram for *CYBB*_pos_B cells. **(C)** Communication network diagram for *CYBB*_neg_B cells. **(D)** Heatmap of differences in outgoing signaling strength between cells. **(E)** Overall interaction strength between specific cell subtypes in the BTLA signaling pathway. **(F)** Bubble plot showing differences in specific ligand-receptor interactions between *CYBB*_pos_B/*CYBB*_neg_B cells (as senders) and immune-related cells.

To elucidate communication mechanisms between different cellular components, we constructed a cell-cell interaction network (Figure 5A). Results indicated extensive interactions among various cell types. The number of interactions between *CYBB*_pos_B and other cells (Figure 5B) was higher than that between *CYBB*_neg_B and other cells (Figure 5C). Further comparison of outgoing signal strength revealed that *CYBB*_pos_B cells exhibited significantly stronger signal output capacity than *CYBB*_neg_B cells (Figure 5D). Comparing signaling pathway characteristics between *CYBB*_pos_B and *CYBB*_neg_B cells revealed that only *CYBB*_pos_B cells could regulate other cells via the BTLA signaling pathway, a capability absent in *CYBB*_neg_B cells (Figure 5E). As signal-sending cells, *CYBB*_pos_B cells mediated intercellular communication through various ligand-receptor pairs. Analysis of interaction counts and ligand-receptor composition showed that these cells primarily established connections with multiple immune cells via the BTLA-TNFRSF14 axis (Figure 5F). Specifically, Macrophages, Tregs, CD8^+^ T cells, CD4^+^ T cells, NK cells, and pDCs interacted with *CYBB*_pos_B cells through this pathway.

### 3.6 Bulk data validation and immune infiltration analysis

**FIGURE 6:**
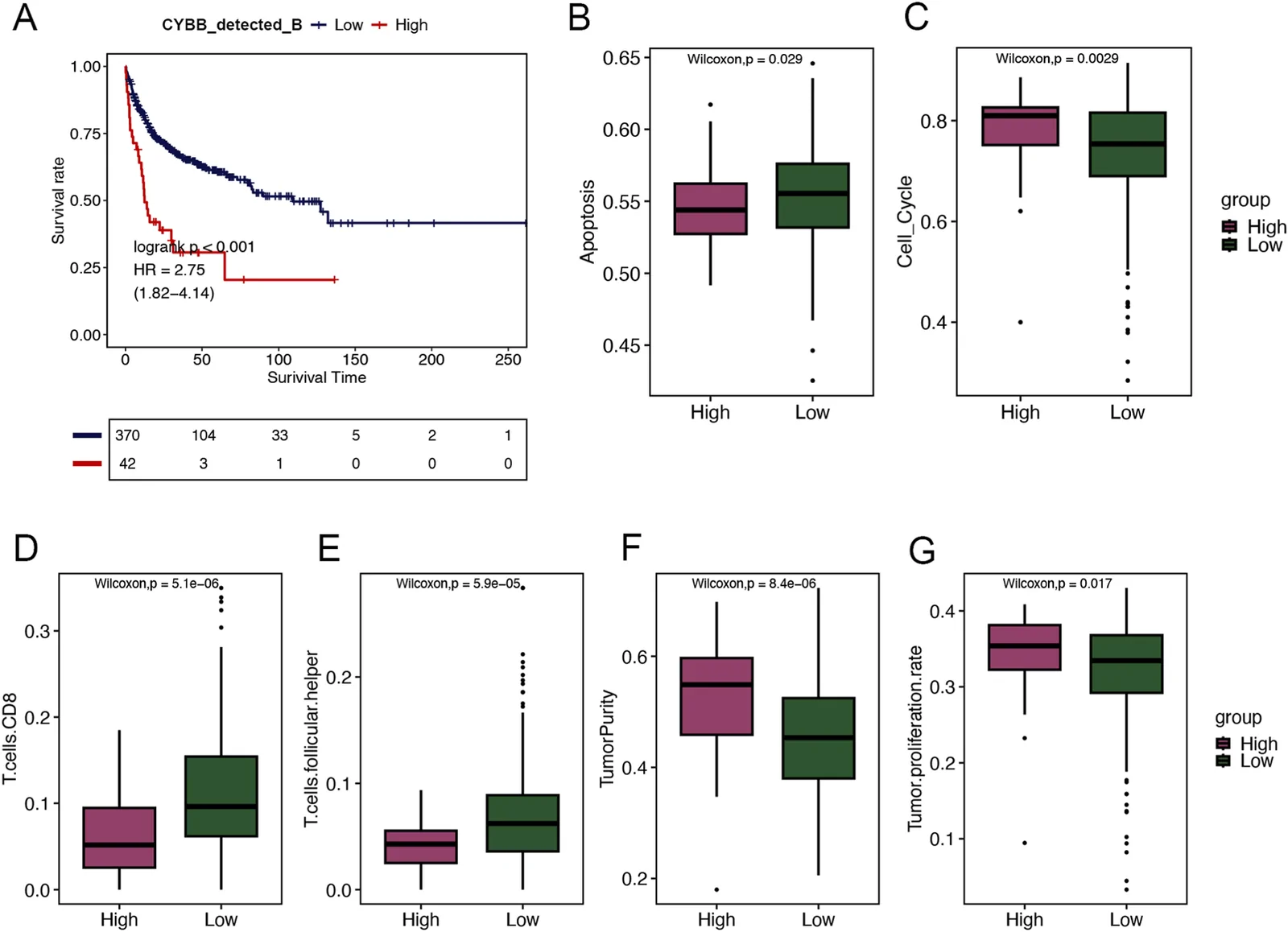
Bulk dataset validation and immune infiltration analysis. Deconvolution analysis of single-cell data was performed to stratify GSE10846 dataset samples into high and low *CYBB* proportion groups. **(A)** Comparison of survival prognosis between the two groups. **(B)** Comparison of apoptosis functional state scores (based on CancerSEA database). **(C)** Comparison of cell cycle functional state scores (based on CancerSEA database). **(D)** Comparison of CD8^+^ T cell infiltration proportions (based on CIBERSORT algorithm). **(E)** Comparison of T follicular helper cell infiltration proportions (based on CIBERSORT algorithm). **(F)** Comparison of tumor purity scores (based on ESTIMATE algorithm). **(G)** Comparison of tumor proliferation activity scores (based on FGE analysis).

To further validate the single-cell analysis results, we mapped single-cell data to the GSE10846 dataset via Bayesian deconvolution, stratifying samples into high-*CYBB* proportion and low-*CYBB* proportion groups. Survival analysis showed that patients in the low-*CYBB* proportion group had a better prognosis (Figure 6A). To validate the prognostic value of *CYBB* expression across multiple independent datasets, we deconvoluted single-cell data to the GSE23501, GSE11318 and GSE181063 datasets, dividing them into high- and low-*CYBB* proportion groups based on optimal cutoffs. Survival analysis indicated that a high *CYBB* proportion was significantly associated with poor patient prognosis (Supplementary Figures S4A–C).

Subsequently, we conducted a systematic tumor microenvironment analysis on the two groups: (1) Scoring based on cancer-related functional states from the CancerSEA database revealed that the low-*CYBB* proportion group had significantly higher apoptosis scores (Figure 6B), while the high-*CYBB* proportion group had significantly higher cell cycle scores (Figure 6C); (2) Evaluation of immune cell subtype infiltration via CIBERSORT showed that the relative proportions of CD8^+^ T cells (Figure 6D) and T follicular helper cells (Figure 6E) were significantly higher in the low-*CYBB* proportion group; (3) Assessment of tumor purity and immune/stromal infiltration using the ESTIMATE algorithm revealed significantly higher tumor purity scores in the high-*CYBB* proportion group (Figure 6F); (4) Functional Gene Enrichment (FGE) analysis characterizing tumor functional features indicated significantly enhanced tumor proliferation activity in the high-*CYBB* proportion group (Figure 6G). Collectively, these results suggest that high *CYBB* expression may drive DLBCL progression by promoting cell cycle progression, enhancing proliferative activity, and shaping an immunosuppressive microenvironment, highlighting its value as a prognostic biomarker and potential therapeutic target in DLBCL.

### 3.7 Functional validation of *CYBB* as a berberine target

**FIGURE 7:**
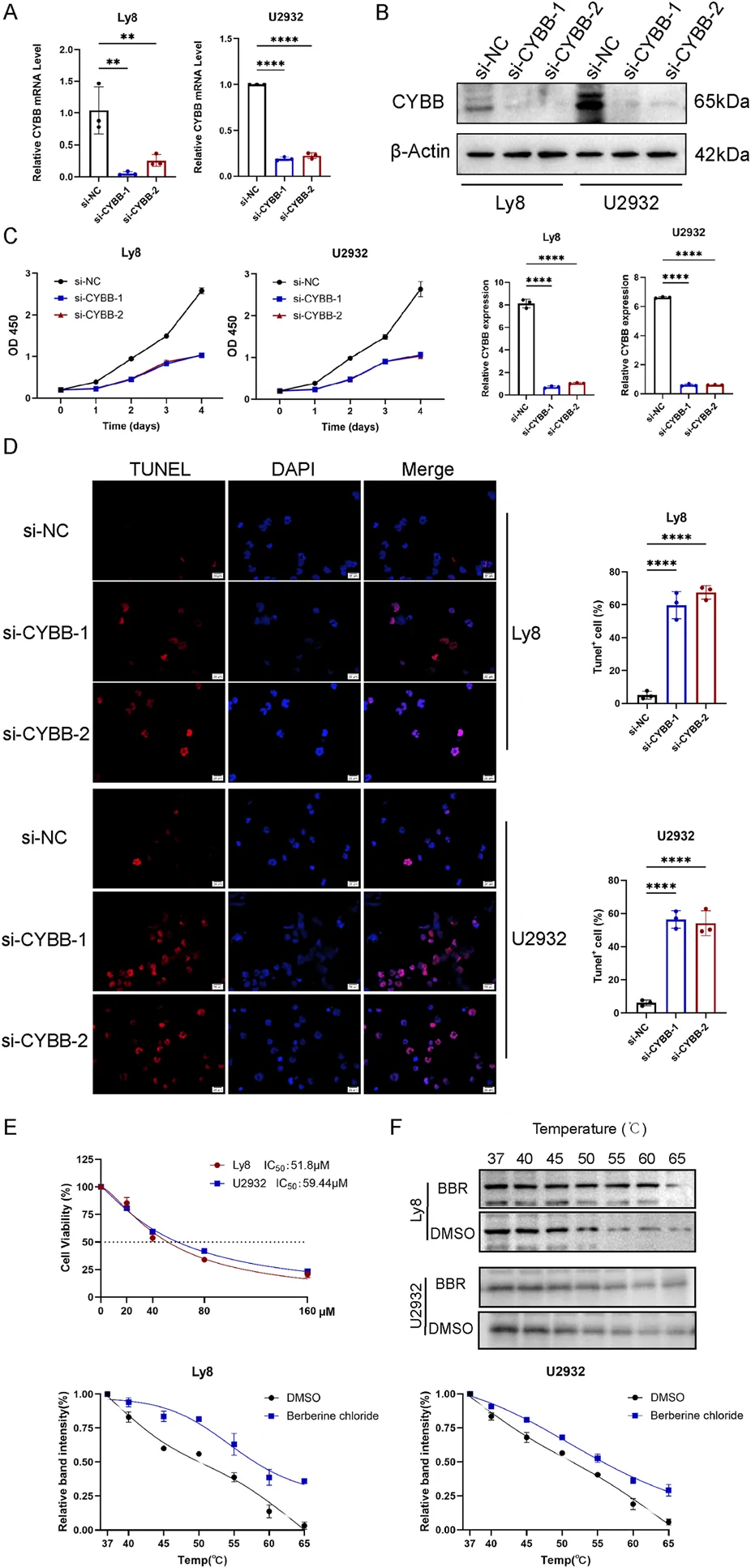
Effects of *CYBB* knockdown and berberine treatment on DLBCL cells. **(A)** Relative mRNA expression levels of *CYBB* in U2932 and Ly8 cells after siRNA knockdown detected by qPCR. **(B)** *CYBB* protein expression in U2932 and Ly8 cells after siRNA-mediated knockdown, as detected by Western blot. β-Actin was used as the loading control/reference protein. **(C)** Effect of *CYBB* knockdown on cell viability detected by CCK-8 assay. The assay was independently repeated five times (n = 5). **(D)** Changes in cell apoptosis induced by *CYBB* knockdown detected by TUNEL assay. TUNEL-positive cells were quantified using ImageJ. **(E)** Dose-dependent changes in cell viability under different concentrations of Berberine treatment. **(F)** Thermal stability of *CYBB* protein after Berberine treatment analyzed by Cellular Thermal Shift Assay (CETSA). CYBB protein levels were compared between berberine-treated and DMSO-treated control samples across the same temperature gradient. The upper and lower CYBB bands represent the berberine and DMSO groups, respectively. Relative CYBB band intensity was quantified to assess changes in CYBB thermal stability after berberine treatment.

To validate the effect of *CYBB* as a berberine target on DLBCL cells, we constructed two siRNAs targeting *CYBB* and transfected them into U2932 and Ly8 cells. qPCR and Western blot results demonstrated significant inhibition of *CYBB* at both mRNA and protein levels in both cell lines, confirming the reliability of the knockdown model (Figures 7A,B). Following the reduction of *CYBB* expression, CCK-8 assays showed a marked decrease in cell viability in both cell lines (Figure 7C). Furthermore, TUNEL assays revealed a significantly increased proportion of apoptotic cells in *CYBB*-knockdown U2932 and Ly8 cells (Figure 7D), suggesting that *CYBB* plays a role in inhibiting apoptosis in DLBCL cells. Berberine treatment experiments indicated a dose-dependent inhibitory effect on both Ly8 and U2932 cells, with IC_50_ values of 51.8 and 59.44 μM, respectively (Figure 7E). To determine whether berberine directly acts on *CYBB*, we performed CETSA and found that berberine treatment significantly enhanced the thermal stability of *CYBB* in both cell lines, with thermal melting curves shifting notably to the right compared to the DMSO control group (Figure 7F). This demonstrates that berberine can directly bind to *CYBB* in living cells, suggesting *CYBB* is a key target for berberine’s action in DLBCL.

## 4 Discussion

In this study, we conducted comprehensive bioinformatic analyses using bulk and single-cell transcriptomics data to characterize berberine-associated molecular programs relate to DLBCL. Together with *in vitro* experimental validation, we prioritized that *CYBB* as a convergent node linking genetic susceptibility, malignant B-cell survival, immune signaling architecture, and pharmacologic response. By linking genetic inference with cell-type–resolved context and pharmacologic perturbation, this work provides an integrated framework for examining how redox-related pathways may intersect with apoptosis resistance, immune regulation, and therapeutic vulnerability in DLBCL.

At the pathway level, MR-prioritized berberine-associated genes were enriched in BCR signaling, MAPK signaling, immune checkpoint pathways, and apoptosis/autophagy programs. Interestingly, these pathway-level findings are highly consistent with established DLBCL biology. It has been reported that chronic active BCR signaling is a central driver of malignant B-cell survival, particularly in activated B-cell–like DLBCL, where it sustains NF-κB- and MAPK-dependent transcriptional programs (Davis et al., 2010; Ngo et al., 2011; Young and Staudt, 2013). Dysregulated MAPK signaling further integrates upstream antigen receptor and Toll-like receptor cues and contributes to proliferation, stress adaptation, and therapeutic resistance (Chapuy et al., 2018; Pasqualucci and Dalla-Favera, 2018; Schmitz et al., 2018). Consistently, our CYBB-centered PPI analysis showed that CYBB–MAPK3 and CYBB–RAC1 were supported by database annotation and experimental evidence, suggesting relatively high-confidence functional associations. MAPK3/ERK1 is biologically relevant to DLBCL because MAPK/ERK activation cooperates with chronic BCR and NF-κB signaling to promote lymphoma-cell survival, c-Myc/Bcl-2-related progression, and drug resistance (Mansoor et al., 2023). Although JUN emerged as a connected node, CYBB–JUN was mainly supported by text-mining evidence and is therefore interpreted cautiously. Nevertheless, JUN/AP-1 has reported DLBCL relevance, particularly in CARD11-activated ABC-DLBCL, where c-Jun and JunB contribute to extracellular-matrix adhesion, invasion, tumor formation, and extranodal dissemination (Blonska et al., 2015). Nevertheless, none of the 21 nominally associated genes survived multiple comparison correction across the 315-gene MR screening, therefore these genes should be interpreted as hypothesis-generating candidates rather than statistically significant causal hits.

Our MR and transcriptomic analyses also highlighted apoptosis-related processes in DLBCL. Apoptotic competence is an important determinant of sensitivity to cytotoxic therapy in aggressive lymphomas, and clinical studies have shown that transcriptional signatures consistent with intact apoptotic machinery are associated with more favorable outcomes (Lenz, Wright, Dave, et al., 2008; Norris et al., 2024). However, transcriptional activation of apoptosis-related pathways does not necessarily correspond to execution of cell death. In many cancers, stress-responsive transcriptional programs may reflect apoptotic priming or compensatory survival states rather than terminal apoptosis (Certo et al., 2006; Fulda, 2010). This distinction is relevant for interpreting our single-cell observations, in which *CYBB*-positive B cells exhibited enrichment of apoptosis- and stress-related transcriptional programs despite *CYBB* being associated with adverse prognosis at the bulk-tissue level. Functional experiments further revealed *CYBB* silencing reduced DLBCL cell viability and increased apoptosis, suggesting that *CYBB* may support a pro-survival, apoptosis-resistant cellular state. These findings are consistent with prior evidence that redox-dependent signaling can uncouple apoptotic pathway engagement from cell death execution, thereby favoring tumor survival, genomic instability, and therapy resistance (Trachootham et al., 2009; Weinberg and Chandel, 2009). Thus, the enrichment of apoptotic transcriptional programs in *CYBB*-positive cells may reflect a stressed but adaptation-capable tumor state rather than effective apoptotic clearance.

*CYBB* encodes gp91phox, the catalytic subunit of the NOX2 NADPH oxidase complex, which is a major source of regulated reactive oxygen species (ROS) in hematopoietic cells (Bedard and Krause, 2007). ROS biology in cancer is inherently context dependent as excessive ROS is cytotoxic, whereas moderate ROS levels function as signaling mediators that promote proliferation, metabolic rewiring, and stress tolerance (Ngo et al., 2011; Weinberg and Chandel, 2009). In hematologic malignancies, NOX2 has been increasingly implicated in adverse disease biology. In acute myeloid leukemia, NOX2 expression and activity have been linked to chemoresistance, leukemic stem cell maintenance, metabolic adaptation, and poor clinical outcomes (de Beauchamp et al., 2022; Jones et al., 2018; Paolillo et al., 2022). Our analyses prioritized *CYBB* within a DLBCL-specific context in which higher expression is consistently associated with poorer prognosis across independent cohorts, higher tumor purity, increased proliferative signatures, and reduced infiltration of cytotoxic immune subsets. Importantly, *CYBB* was advanced for downstream analyses not solely on the basis of nominal MR evidence, but because it showed convergent support from transcriptomic validation, prognostic analyses, single-cell characterization, structural prediction, and functional experiments. These associations suggest that NOX2-dependent redox signaling may similarly support aggressive tumor behavior in lymphoma. Together with the knockdown experiments, these findings suggest that CYBB may contribute to DLBCL development by promoting malignant B-cell survival and limiting apoptosis, potentially through NOX2-related redox signaling. However, we did not directly measure NOX2 enzymatic activity, superoxide production, or ROS generation after berberine treatment. Therefore, whether berberine functionally inhibits CYBB/NOX2 activity remains to be established. Future studies incorporating NOX2 activity assays, ROS/superoxide measurements, and CYBB rescue or re-expression experiments will be needed to define the functional role of CYBB in berberine-induced growth suppression in DLBCL cells. At the same time, NOX2 signaling has important immune-regulatory roles in non-malignant settings. In B cells, NOX2-derived ROS modulate endosomal trafficking and restrain excessive Toll-like receptor signaling, thereby shaping NF-κB activation and inflammatory responses (Gordon et al., 2024; Liu et al., 2024). These observations underscore the pleiotropic nature of NOX2, with functional consequences that depend on cellular context and signaling balance. Our data suggest that, in malignant B cells, *CYBB* expression is associated with a state in which redox-related signaling may align with survival and immune modulation rather than immune restraint. Of note, DLBCL is biologically heterogeneous (Schmitz et al., 2018), and whether CYBB expression is preferentially associated with specific molecular subtypes remains unresolved. In our *in vitro* models, CYBB protein expression was detected in both OCI-Ly8 cells, representing the GCB subtype, and U2932 cells, representing the ABC subtype, suggesting that CYBB expression is not restricted to a single commonly used subtype model. However, these cell-line findings do not establish pan-subtype relevance in patient tumors. Further analyses in well-annotated cohorts will be needed to determine whether CYBB expression tracks with GCB, ABC, or LymphGen-defined genetic subtypes.

Beyond tumor-intrinsic features, our single-cell and cell–cell communication analyses indicate that *CYBB*-positive B cells reside within a distinct immunoregulatory milieu. *CYBB*-positive B cells displayed increased outgoing signaling capacity and selectively engaged the BTLA–TNFRSF14 (HVEM) axis in interactions with multiple immune cell types. The BTLA–HVEM pathway is a well-characterized immune checkpoint circuit that negatively regulates T-cell activation, cytokine production, and effector function (Pasero et al., 2012; Sedy et al., 2005; Watanabe et al., 2003). Genetic and functional studies have shown that perturbation of HVEM–BTLA signaling alters T-cell help to germinal center B cells and can contribute to lymphomagenesis, and recurrent alterations in TNFRSF14 are frequent in germinal center–derived lymphomas and are associated with immune escape and adverse outcomes (Boice et al., 2016; Carreras et al., 2019; Mintz et al., 2019). Moreover, recent evidence indicates that BTLA–HVEM signaling can limit the efficacy of T-cell–based immunotherapies, including CAR T-cell treatment (Guruprasad et al., 2024). Our results extend this literature by directly linking checkpoint-oriented immune communication to a *CYBB*-defined malignant B-cell state. In contrast, *CYBB*-negative B cells preferentially communicated with natural killer (NK) cells through the BAG6–NCR3 (NKp30) axis. BAG6 was originally identified as a stress-induced ligand that activates NKp30-mediated cytotoxicity and promotes immune surveillance (Pogge von Strandmann et al., 2007). Subsequent studies demonstrated that soluble or vesicle-associated BAG6 can impair NKp30-dependent NK-cell function, thereby facilitating immune evasion in B-cell malignancies (Binici et al., 2013; Groh et al., 2006; Reiners et al., 2013). These findings indicate that BAG6–NKp30 signaling represents a finely balanced immunoregulatory module. Our analyses are consistent with a model in which *CYBB*-negative B-cell states may be more permissive to NK-mediated surveillance, whereas *CYBB*-positive states are associated with checkpoint-dominated immune regulation.

A major strength of this study is that we leveraged evidence from observational multi-omics integrative analyses and experimental validation supporting *CYBB* as a functionally relevant target of berberine in DLBCL. Molecular docking and molecular dynamics simulations suggested stable, energetically favorable binding of berberine within the *CYBB* active pocket, while cellular thermal shift assays supported berberine–*CYBB* target engagement in DLBCL cells (Martinez Molina et al., 2013). These findings extend prior reports that berberine modulates NF-κB signaling and immune checkpoints (Fu et al., 2013; Haftcheshmeh et al., 2022; Ren et al., 2021) by identifying a tumor-intrinsic redox regulator as a putative cellular target engaged by berberine. In relation to the previously reported c-MYC/CD47 immune-evasion axis, *CYBB* showed only weak positive correlations with *CD47* and *c-MYC* expression, suggesting partial but limited overlap. Thus, *CYBB*-related mechanisms may mainly reflect apoptosis regulation, redox signaling, and immune microenvironment remodeling rather than direct dependence on the *c-MYC/CD47* axis. Further ChIP, promoter, and perturbation analyses are needed to test potential regulatory links. Functionally, both *CYBB* silencing and berberine treatment reduced DLBCL cell viability and promoted apoptosis, supporting a model in which berberine may partially interfere with *CYBB*-associated survival programs. However, these data do not establish *CYBB* as the exclusive mediator of berberine activity or prove direct inhibition of *CYBB* enzymatic function. Moreover, the *in vitro* IC50 values and CETSA concentration exceed reported plasma concentrations after oral berberine administration, which are typically below 0.5 μM. Although berberine may achieve higher tissue than plasma exposure (Han et al., 2021; Liu X.et al., 2022), direct evidence for pharmacologically relevant accumulation in lymphoid tissues or DLBCL lesions remains limited. Therefore, these findings should be interpreted as proof-of-concept evidence rather than direct evidence of clinical feasibility. Future studies using *in vivo* DLBCL models, lymphoma-relevant pharmacokinetic assessment, improved delivery strategies such as nanoformulations or targeted delivery, and combination approaches will be needed to determine the translational relevance of the berberine–CYBB axis. Together, these findings refine current understanding of berberine’s potential mechanisms of action and support further investigation of CYBB/NOX2 as a biologically relevant redox-associated vulnerability in DLBCL. Its clinical prognostic value, however, remains exploratory and will require validation against established risk models such as IPI and CNS-IPI in well-annotated cohorts.

Several limitations should be acknowledged. First, MR sensitivity analyses, including directional consistency, heterogeneity, pleiotropy, leave-one-out analysis, F-statistics, and Steiger filtering, improved the robustness of MR interpretation but did not replace formal multiple-testing correction. Meanwhile, MR also leverages genetically proxied expression and may not fully capture tumor-specific regulatory dynamics shaped by somatic alterations and microenvironmental pressures (Davey Smith and Hemani, 2014). Given the exploratory nature of MR analyses in target genes screening, the identified genes should be interpreted as MR-prioritized candidate genes rather than statistically significant causal hits. In addition, the eQTL instruments were derived from peripheral whole blood and may not fully capture regulatory effects in germinal-center B cells or malignant DLBCL cells. Colocalization analysis did not provide strong evidence that the CYBB eQTL and DLBCL GWAS signals shared the same causal variant, further supporting a careful interpretation of the MR findings. Therefore, these results should be considered hypothesis-generating and require further validation using disease- and cell-type-specific eQTL resources, as well as in-depth experimental studies. Nevertheless, the concordance of MR findings with tumor transcriptomics, survival analyses, and functional perturbation provides strong contextual validation. Another limitation is that molecular subtype annotations were not consistently available across the bulk and single-cell datasets; therefore, we could not rigorously evaluate CYBB expression across GCB, ABC, or LymphGen genetic subtypes. Although CYBB was detectable in both GCB-like OCI-Ly8 and ABC-like U2932 cells, the subtype specificity of CYBB in patient-derived DLBCL remains to be defined. Additionally, while our ligand–receptor analyses implicate BTLA–HVEM and NKp30-related pathways, direct experimental interrogation of these interactions will be required to establish causality *in vivo*. Finally, while our data support the berberine–*CYBB* axis as a candidate mechanism, they do not directly establish that berberine inhibits *CYBB* enzymatic activity or that *CYBB* is the sole mediator of berberine-induced growth inhibition. Although temperature-gradient CETSA provided initial evidence of berberine–CYBB target engagement, ITDRF-CETSA was not performed; therefore, concentration-dependent target engagement was not directly demonstrated. This is particularly relevant given the polypharmacological nature of berberine. In addition, the *in vitro* berberine concentrations used for cell-viability and CETSA assays exceed commonly reported plasma concentrations after oral administration. Given the pleiotropic nature of berberine, future studies incorporating NOX2 activity assays, ROS measurements, *CYBB* rescue or overexpression experiments, and binding-deficient *CYBB* mutants will be needed to define the specificity of this axis and quantify the relative contribution of *CYBB* to berberine’s overall anti-tumor effects.

In summary, this study identifies *CYBB* as a genetically supported and functionally relevant node associated with berberine-linked molecular programs in DLBCL. By integrating causal inference, multi-omics profiling, structural modeling, and functional assays, our findings suggest that *CYBB* is associated with malignant B-cell survival, apoptosis resistance, and features of immune microenvironmental remodeling. These results refine current understanding of berberine’s potential mechanisms of action and support further investigation of *CYBB*/NOX2 as a prognostic marker and therapeutic vulnerability in DLBCL, particularly in strategies that combine redox modulation with apoptosis- and immune-based interventions.

## Data Availability

The original contributions presented in the study are included in the article/Supplementary Material, further inquiries can be directed to the corresponding authors.

## Supplementary material

SUPPLEMENTARY FIGURE S1 Mendelian Randomization Analysis. **(A)** Forest plot showing the overall MR effect of individual SNPs on DLBCL risk. Each horizontal line represents an independent SNP; the red line indicates the pooled effect size. **(B)** Funnel plot assessing potential data bias; symmetrical distribution of data points suggests minimal bias in this study. **(C)** Leave-one-out sensitivity analysis demonstrating the stability of the overall effect size by sequentially excluding each SNP to ensure no single SNP disproportionately influences the results. **(D)** Scatter plot displaying the relationship between SNPs and DLBCL risk.

SUPPLEMENTARY FIGURE S2 Kaplan-Meier Survival Curves Stratified by High/Low Expression Levels of Berberine Target Genes.

SUPPLEMENTARY FIGURE S3 Construction and Application of Machine Learning-Based DLBCL Survival Prediction and Risk Stratification Models. **(A)** Heatmap and bar chart showing the performance comparison of 101 machine learning models for DLBCL survival prediction (including single algorithms and combined models). Model performance is measured by the C-index; darker colors in the heatmap represent higher performance. The bar chart presents the average C-index of each model across all datasets in descending order. **(B-E)** Risk stratification results based on the optimal prognostic model: DLBCL patients were divided into high-risk and low-risk groups, followed by detailed analysis combined with key clinical prognostic factors (disease stage, IPI score, age).

SUPPLEMENTARY FIGURE S4 Bulk Dataset Survival Validation. **(A)** Survival analysis of high and low *CYBB* proportion groups in the GSE23501 dataset. **(B)** Survival analysis of high and low *CYBB* proportion groups in the GSE11318 dataset. **(C)** Survival analysis of high and low *CYBB* proportion groups in the GSE181063 dataset.
